# Peripheral Blood Immune Profiling of Convalescent Plasma Donors Reveals Alterations in Specific Immune Subpopulations Even at 2 Months Post SARS-CoV-2 Infection

**DOI:** 10.3390/v13010026

**Published:** 2020-12-25

**Authors:** Nikolaos Orologas-Stavrou, Marianna Politou, Pantelis Rousakis, Ioannis V. Kostopoulos, Ioannis Ntanasis-Stathopoulos, Edison Jahaj, Eleni Tsiligkeridou, Maria Gavriatopoulou, Efstathios Kastritis, Anastasia Kotanidou, Meletios-Athanasios Dimopoulos, Ourania E. Tsitsilonis, Evangelos Terpos

**Affiliations:** 1 Department of Biology, School of Sciences, National and Kapodistrian University of Athens, 15784 Athens, Greece; norologas@med.uoa.gr (N.O.-S.); rousakisp@gmail.com (P.R.); gikosto@gmail.com (I.V.K.); rtsitsil@biol.uoa.gr (O.E.T.); 2 Hematology Laboratory-Blood Bank, Aretaieion Hospital, School of Medicine, National and Kapodistrian University of Athens, 11528 Athens, Greece; mariannapolitou@gmail.com; 3 Department of Clinical Therapeutics, Alexandra General Hospital, School of Medicine, National and Kapodistrian University of Athens, 11528 Athens, Greece; johnntanasis@med.uoa.gr (I.N.-S.); eleni.tsili@yahoo.com (E.T.); mariagabria@gmail.com (M.G.); ekastritis@gmail.com (E.K.); mdimop@med.uoa.gr (M.-A.D.); 4 First Department of Critical Care Medicine and Pulmonary Services, Evangelismos General Hospital, National and Kapodistrian University of Athens, 11527 Athens, Greece; edison.jahaj@gmail.com (E.J.); akotanid@med.uoa.gr (A.K.)

**Keywords:** SARS-CoV-2, COVID-19, convalescent plasma donors, immune profiling

## Abstract

Immune profiling of patients with COVID-19 has shown that SARS-CoV-2 causes severe lymphocyte deficiencies (e.g., lymphopenia, decreased numbers, and exhaustion of T cells) and increased levels of pro-inflammatory monocytes. Peripheral blood (PB) samples from convalescent plasma (CP) donors, COVID-19 patients, and control subjects were analyzed by multiparametric flow cytometry, allowing the identification of a wide panel of immune cells, comprising lymphocytes (T, B, natural killer (NK) and NKT cells), monocytes, granulocytes, and their subsets. Compared to active COVID-19 patients, our results revealed that the immune profile of recovered donors was restored for most subpopulations. Nevertheless, even 2 months after recovery, CP donors still had reduced levels of CD4^+^ T and B cells, as well as granulocytes. CP donors with non-detectable levels of anti-SARS-CoV-2-specific antibodies in their serum were characterized by higher Th9 and Th17 cells, which were possibly expanded at the expense of Th2 humoral immunity. The most noticeable alterations were identified in previously hospitalized CP donors, who presented the lowest levels of CD8^+^ regulatory T cells, the highest levels of CD56^+^CD16^−^ NKT cells, and a promotion of a Th17-type phenotype, which might be associated with a prolonged pro-inflammatory response. A longer follow-up of CP donors will eventually reveal the time needed for full recovery of their immune system competence.

## 1. Introduction

COVID-19, the disease caused by the Severe Acute Syndrome Coronavirus (SARS-CoV)-2, is a potentially fatal disease with approximately 20% of patients experiencing severe symptoms, 14% developing respiratory distress requiring supplemental oxygen, and 5% requiring hospitalization in Intensive Care Units (ICUs). Although it affects multiple organs leading to multi-systemic failure and often to death [1], COVID-19 is apparently related to excessive host inflammatory responses and thus, it looks more similar to a disease of the immune system [2]. Still, it is elusive how this new virus evades the immune system and escapes hosts’ immune surveillance, although some delineated pathways of invasion may be similar to mechanisms used by other coronaviruses [3].

Under the pressure to promptly reveal biomarkers predicting the severity of COVID-19 and thus assisting the clinical management of hospitalized patients, numerous peripheral blood cells and factors have been analyzed. Severe COVID-19 cases have been associated with significant alterations in immune cell subsets both quantitatively and functionally. Although studies to date were performed at diverse patient cohorts, they converge at the conclusion that COVID-19 patients exhibit early upon infection deficient production of interferons (IFNs) types I and III and increased serum levels of pro-inflammatory cytokines (interleukin (IL)-6, IL-1β, tumor-necrosis factor (TNF)) in contrast to the classical anti-viral immune response mounted against other coronaviruses [4]. Consequently, pronounced lymphopenia (i.e., low numbers of CD3^+^ and CD4^+^ T cells, particularly of the Th1-type and regulatory T cells (Tregs); very low numbers of CD8^+^ T, B and natural killer (NK) cells) and T and NK cell exhaustion (e.g., reduced expression of CD26; increased expression of T-cell immunoglobulin and mucin domain-3 (TIM-3) and programmed cell death protein (PD)-1) have been recorded [5,6,7,8]. Immune system deterioration is further exacerbated by the presence of increased percentages of myeloid-derived suppressor cells (MDSCs) and inflammatory monocytes (CD14^+^CD16^+^) [9,10,11]. The end result is that the immune system particularly of severe COVID-19 patients fails to generate a robust response to SARS-CoV-2 and accordingly eliminate infection. 

To date, specific therapeutic interventions for COVID-19 are not available. The treatment of severely ill patients with anti-retrovirals (lopinavir, ritonavir, ribavirin, remdesivir), anti-inflammatory drugs (corticosteroids), monoclonal antibodies (mAbs) (tocilizumab, bevacizumab), immunomodulators (IFN-α, IFN-β, fingolimod), and combinations thereof has not been able to fully reverse fatal outcomes [12,13]. A proposed adjunctive therapy is the transfusion of convalescent plasma (CP), particularly in critically ill COVID-19 ICU patients [13,14]. CP therapy has been administered for years to treat patients with various infectious diseases including those caused by SARS, MERS, Ebola, and influenza, and clinical results supported its neutralizing properties. Moreover, apart from the direct antibody (Ab)-mediated antiviral effect, CP contains anti-inflammatory cytokines, natural antibodies, and antimicrobial proteins such as defensins and pentraxins, which can control over-activation of the immune system and act in an immunomodulatory mode [15]. In the case of SARS-CoV-2 and in support of the beneficial use of CP, a recent meta-analysis showed that CP administration was associated with a significant reduction in mortality [16], whereas the transfusion of CP in 20,000 COVID-19 patients proved that it is safe and carries a very low risk of complications [17]. Nevertheless, in a most recent clinical trial, CP infusion in 228 COVID-19 patients with severe pneumonia did not offer any significant benefit as for clinical status and overall mortality rate [18].

Although many reports have analyzed in detail immune responses in the course of COVID-19, there is limited knowledge regarding possible alterations in immune cells that may persist in recovered individuals after SARS-CoV-2 infection. CP is collected by apheresis from patients who have established a humoral immune response against SARS-CoV-2 following COVID-19 resolution [15]; therefore, CP donors are the ideal cohort for immune profiling after recovery. In the present study, we assessed peripheral blood (PB) immune cell signatures in CP donors in an effort to elucidate if the immune system is fully competent after recovery, both numerically and functionally. Our study may provide important information on phenotypic immune signatures persisting after SARS-CoV-2 infection, highlight differences between asymptomatic, mild, and severe COVID-19 cases and eventually facilitate the recognition of Ab-negative SARS-CoV-2-infected individuals, who may have developed a competent anti-viral cell-mediated immune response and thus could be protected from reinfection.

## 2. Materials and Methods 

### 2.1. Donors, Clinical Characteristics, and Detection of Anti-SARS-CoV-2 Antibodies

Donors included in the study (*n* = 95) were divided into 5 groups according to their clinical characteristics. Candidate donors underwent a blood donors’ history questionnaire and symptoms related to SARS-Co-V infection were also recorded (i.e., fever, fatigue, headache, cough, dyspnea, diarrhea, loss of smell, loss of taste, duration of symptoms). For the detection of anti-SARS-CoV-2 Abs, commercially available ELISAs (Euroimmun Medizinische Labordiagnostika AG, Lubeck, Germany), which detect plasma IgG and IgA antibodies against the recombinant spike protein (S1 domain) of SARS-CoV-2 were used according to the manufacturer’s instructions. Ratios < 0.8 were considered negative, ≥0.8 to <1.1 were considered borderline, and ≥1.1 were considered positive.

### 2.2. Blood Sample Collection and Staining

PB samples were collected through venipuncture in 2 mL BD Vacutainer^®^ spray-coated K_2_EDTA blood collection tubes (BD Biosciences, San Jose, CA, USA; #367841). One hundred μL of whole blood was transported into 5 mL Corning™ Falcon™ Round-Bottom Polystyrene Tubes (#352054) and stained with 12 monoclonal antibodies against the surface markers: CD45-APC-H7 (clone 2D1, #560178); CD3-PerCP (clone SK7, #340663); CD4-BV510 (clone SK3; #562970); CD8-PE (clone HIT8a, #555635); CD14-BV605 (clone M5E2, #564054); CD16-APC (clone B73.1, #561304); CD56-APC-R700 (clone NCAM16.2, #565139); CD25-PE-CF594 (clone M-A25, #562403); CD11b-BV786 (clone D12, #742642); CD183 (CXCR3)-BV421 (clone 1C6/CXCR3, #562558); CD194 (CCR4)-BV650 (clone 1G1, #744140); and CD196 (CCR6)-BB515 (clone 11A9, #564479) (all from BD Biosciences). The staining procedure was performed in BD Horizon™ Brilliant Stain Buffer (BD Biosciences, #563794) followed by red blood cells lysis with 1x BD FACS™ lysing solution (BD Biosciences, #349202) as suggested by the manufacturer for the Lyse-no-Wash protocol.

### 2.3. Sample Preparation for Flow Cytometry Analysis

Samples were run on a three-laser (blue, red, violet) 12-fluorochrome 14-parameter BD FACSCelesta (BD Biosciences). The initial set-up of PMT voltages was performed with unstained control donor cells, while the appropriate compensation set-up was effectuated with multiple single-tube staining of all markers/fluorophores used, utilizing the BD™ CompBeads Set Anti-Mouse Igκ (BD Biosciences, #552843). A total number of 100,000 white blood cells (WBCs) were acquired per sample, with the threshold set at the CD45-APC-H7 channel to avoid measurement of cell debris events, due to the Lyse-no-Wash protocol followed for sample preparation.

### 2.4. Flow Cytometry Gating Strategy

Data analysis was performed with the BD FACSDiva^TM^ software (BD Biosciences). The gating strategy followed, allowed for the initial selection of CD45^+^ cells (WBC region), which were further separated into three main gates on a CD45/side scatter (SSC) dot plot corresponding to lymphocytes, monocytes, and granulocytes (Figure S1A). Lymphocytes were further represented on a CD3 vs. SSC-A plot, which allowed the selection of CD3^+^ T cells. The CD4 vs. CD8 expression pattern of gated CD3^+^ T cells was represented on a dot plot, where subpopulations were selected from the CD4^+^ and CD8^+^ T cells. Each was further plotted vs. CD25, allowing gating of CD25^high^CD4^+^ regulatory T cells (Tregs) and CD25^high^CD8^+^ Tregs. To analyze polarized helper T cell (Th) subpopulations, CD3^+^CD4^+^ T cells were separately represented on plots vs. CD183, CD194, and CD196. Accordingly, on each plot, the Th1, Th2, and Th9 subpopulations were gated, respectively. The Th17 subpopulation was estimated on CD3^+^CD4^+^ T cells that coexpressed CD194 and CD196; the percentages of Th17 cells were obtained through the Boolean approach. The CD3-negative (-) cells were represented on a CD16 vs. CD56 plot, and NK cell subpopulations were gated; B cells were gated on the same plot and were estimated indirectly being mutually negative for T cell and NK cell markers (CD3^−^CD16^−^CD56^−^). To acquire the percentage of NKT cells, CD3^+^CD4^+^/CD8^+^ cells were represented on a CD16 vs. CD56 dot plot. Monocyte subpopulations were gated on a CD14 vs. CD16 dot plot, in which the monocyte region from the initial CD45 vs. SSC-A plot was represented. The population hierarchy tree (Figure S1B) allowed for the concomitant determination of the percentages of 24 phenotypically distinct (Figure S1C for Th1, Th2, Th9, and Th17) PB immune cell subpopulations.

### 2.5. Statistical Analysis 

Data were analyzed using GraphPad Prism v. 7 software (San Diego, CA, USA). Results are expressed as means ± standard deviation (SD). For statistical analysis, one-way ANOVA followed by Holm–Sidak post-hoc tests were performed. *p*-values < 0.05 were considered statistically significant. Differences among groups were compared with Wilcoxon’s signed-rank test.

## 3. Results

A total of 95 individuals were included in the study. Of these, 10 were control donors who reported no symptoms, tested negative with RT-qPCR for SARS-CoV-2, and were also negative for the presence of anti-SARS-CoV-2 IgGs and IgAs (control group). Of the SARS-CoV-2-infected donors, 74 individuals reported previous SARS-CoV-2 infection and voluntarily consented to donate CP. The median time from the onset of symptoms or a positive PCR for SARS-Co-V (whatever first) was 61.5 days (range 26–100). These donors were divided in 3 groups according to PCR and IgG/IgA evaluation, and/or severity of resolved COVID-19. In particular, 12 individuals had a positive PCR test but no measurable humoral response for anti-SARS-CoV-2 IgGs and/or IgAs (PCR+ Ab−); 38 PCR-positive (+) individuals had detectable IgG and/or IgA anti-SARS-CoV-2 Abs but were not hospitalized (mild COVID-19 cases; PCR+ Ab+ non-hospitalized) and 24 individuals who were positive for both PCR and anti-SARS-CoV-2 Abs had required hospitalization during the course of COVID-19 (severe COVID-19 cases; PCR+ Ab+ hospitalized). Finally, 11 patients were hospitalized with active COVID-19 at the time of blood collection. Details on the demographic and clinical characteristics of all participants are shown in Table 1.

PB lymphocytes (T, B, NK, and NKT cells), monocytes, granulocytes, and their subpopulations were gated following a uniform strategy (Figure S1A). Thus, we were able to evaluate the percentages of 24 phenotypically distinct populations for all groups of donors (Table 2 and Figure S1B). We also estimated the ratios CD4^+^/CD8^+^ T cells, Th1/Th2, Th1/Th17, Th9/Th17, and Th17/Treg polarized T cells, as well as the ratios of mature/memory-like NK cells, classical/non-classical monocytes, granulocytes/lymphocytes, granulocytes/CD3^+^ T cells, and CD11b^+^/CD11b^−^ granulocytes, as some of these ratios were reported to predict the outcome of COVID-19 patients [19,20]. 

**Figure 1:**
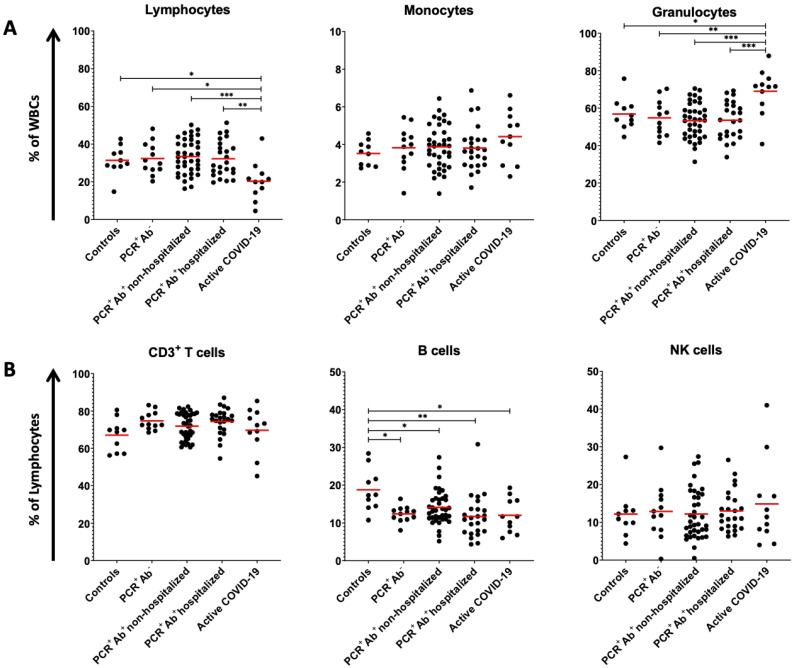
Distribution of (**A**) lymphocytes, monocytes, and granulocytes as percentage of white blood cells (WBCs) and (**B**) CD3^+^ T, B, and NK cells as percentage of lymphocytes in healthy donors (controls), the three groups of recovered CP donors and active COVID-19 patients. Each point represents the percentage determined for each single donor or patient. Bars show mean values. *, *p* < 0.05; **, *p* < 0.01; ***, *p* < 0.001(one-way ANOVA; post-hoc analysis by Holm–Sidak test).

Each subset, regardless of donors’ age and sex, displayed great variability in its relative abundance to the total immune profile analyzed, although clear differences could be identified. We first analyzed the major PB populations (i.e., lymphocytes, monocytes, and granulocytes) and noticed that mean percentages of lymphocytes and granulocytes differed significantly (ranging in statistical significance from *p* < 0.05 to *p* < 0.001) between active COVID-19 patients and the three groups of recovered CP donors and controls (Figure 1A). Monocytes were also higher in active COVID-19 patients, but their difference with the other groups did not reach statistical significance. Lymphocyte subsets (CD3^+^ T cells and NK cells) revealed marginal differences in their percentages among groups; on the contrary, B cells were decreased in all groups compared with controls and statistically significant differences were observed for the groups of active COVID-19 patients (11.97% vs. 18.74% of controls; *p* < 0.05), non-hospitalized and hospitalized CP donors (14.07% (*p* < 0.05) and 11.66% (*p* < 0.01), respectively) and recovered individuals that were PCR+ but had no detectable levels of Abs (12.35%; *p* < 0.05) (Figure 1B).

To test the performance of our 12-color Ab panel, we next looked at differences in specific immune subpopulations between active COVID-19 patients and controls. Active COVID-19 patients showed a characteristic immune profile (Figure S2 and Table 3), with a lower percentage of lymphocytes (20.31% vs. 31.41% of controls; *p* < 0.05); CD4^+^ Tregs and CD8^+^ Tregs were also lower in the active COVID-19 group, but only differences for CD4^+^ Tregs were statistically significant (0.86% vs. 2.05% of controls; *p* < 0.001). Of the T helper cell compartment, Th1-type cells were low (*p* < 0.001) and Th2-type cells were high (*p* = 0.001), leading to a reduced Th1/Th2 ratio (1.19 vs. 6.46 of controls; *p* < 0.001); Th17-type cells were higher in active COVID-19 patients and Th9 marginally increased; however, the ratios Th1/Th17 and Th9/Th17 (*p* < 0.05) were both very low, whereas the Th17/CD4^+^ Treg ratio was increased, suggesting a differential Th cell polarization between active COVID-19 patients and controls. Of the NK cell subsets, percentages of immature and mature NK cells did not differ, but memory-like NK cells were significantly lower in active COVID-19 patients (1.44% vs. 2.61% of controls; *p* < 0.01), leading to a 6-fold mean increase in the mature/memory-like NK cell ratio in the active COVID-19 group (19.47 vs. 3.55 of controls; *p* < 0.001). Although NKT cells are generally of low abundance in the PB, their percentages were also found increased in active COVID-19 patients; the CD3^+^ CD56^+^ CD16^−^ compartment by ≈2-fold (4.22% vs. 1.80% of controls; *p* < 0.001) and the CD3^+^ CD56^−^ CD16^+^ compartment by ≈3-fold (0.64% vs. 0.19% of controls). Regarding monocytes, a significant higher prevalence of intermediate monocytes in active COVID-19 patients was observed (6.57% vs. 0.81% of controls; *p* < 0.001) with respective lower percentages of classical (86.25% vs. 95.04% of controls; *p* < 0.05) and higher percentages of non-classical monocytes. Total granulocytes and their activated CD11b^+^ subset were also increased in COVID-19 patients (69.0% vs. 56.84%, *p* < 0.001 and 72.33% vs. 46.12% of controls, respectively) at the expense of a statistically significant downregulation of the CD11b^−^ granulocyte counterpart (15.74% vs. 47.29% of controls; *p* < 0.05). Accordingly, the ratios of granulocytes/lymphocytes and granulocytes/CD3^+^ T cells were highly increased, and in both cases, the differences were statistically significant (5.16 vs. 2.02 of controls, *p* < 0.01 and 7.19 vs. 3.14 of controls, *p* < 0.01, respectively). Taken together, although the number of active COVID-19 patients included in this study was limited (*n* = 11), the phenotypic changes in their WBC subpopulations detected herein are in agreement with published reports [21,22,23,24] and support the severe deficiency of both innate and adaptive immune responses in hospitalized patients with active COVID-19.

**Figure 2:**
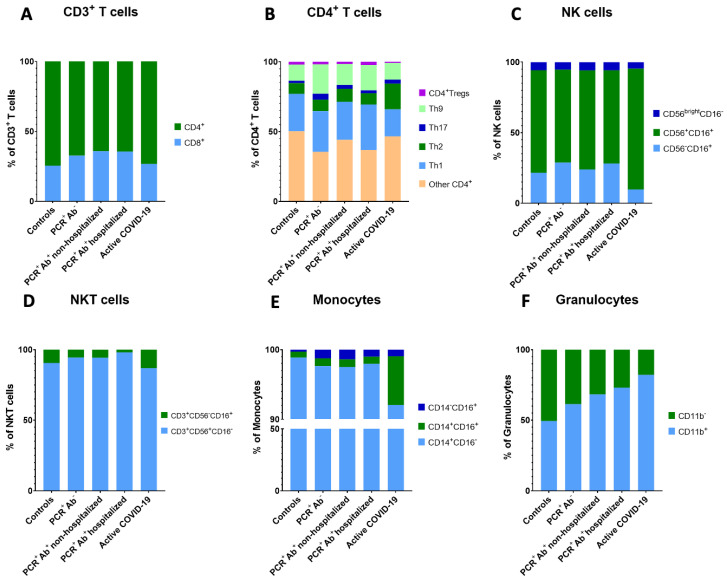
Distribution of (**A**) CD4^+^ and CD8^+^ subpopulations in CD3^+^ T cells, (**B**) Th1, Th2, Th9, Th17, and Treg subpopulations in CD4^+^ T cells, (**C**) immature (CD56^bright^CD16^−^), mature (CD56^+^CD16^+^), and memory-like (CD56-CD16^+^) subpopulations in NK cells, (**D**) CD3^+^CD56^+^CD16^−^ and CD3^+^CD56^−^CD16^+^ subpopulations in NKT cells, (**E**) classical (CD14^+^CD16^−^), intermediate (CD14^+^CD16^+^), and non-classical (CD14-CD16^+^) subpopulations in monocytes, and (**F**) CD11b+ and CD11b^−^ subpopulations in granulocytes in controls, the three groups of recovered CP donors and active COVID-19 patients.

We next considered particular differences in the immune profile between controls and CP donors. Although percentages of most cell subsets were restored to the levels of controls at the time of CP donation (ca. 2 months after recovery), specific alterations were observed in the levels of CD4^+^ (lower) and CD8^+^ (higher) T cells in CP donors who developed Abs and recovered from mild (non-hospitalized) or severe (hospitalized) COVID-19 (60.11% and 60.75%, respectively, compared to 71.36% of controls for CD4^+^, *p* < 0.01; 33.49% in both groups compared to 24.38% of controls for CD8^+^, *p* < 0.05). In CP donors that did not develop Abs, CD4^+^ and CD8^+^ T cell percentages did not differ significantly from controls (64.11% and 31.02%, respectively; Figure 2A). However, in all CP donors’ groups, a ca. 2-fold reduced CD4^+^/CD8^+^ T cell ratio was recorded (*p* < 0.05), which is an observation that in conjunction with the lower percentages of CD8^+^ Tregs suggests that the prolonged presence of CD8^+^ effectors is probably still necessary to modulate the anti-viral response.

Analysis of CD4^+^ T cell subsets revealed the restoration of Th1-, Th2-type, and CD4^+^ Tregs in all groups of CP donors (Figure 2B), and a Th17- and Th9-polarized immunity in recovered individuals that did not generate Abs (4.22% vs. 1.73% of controls for Th17; 21.08% vs. 11.57% of controls for Th9). Accordingly, the Th9/Th17 ratio was highly increased in this group (41.82 vs. 14.33 of controls); since Th9 cells promote the Th17 phenotype, this skewed immune response could be associated with the persistence of a generalized inflammatory reaction (mediated by Th17 cells) being more intense in the lung (mediated by Th9 cells) of Ab- recovered individuals. What was also noticed in the same group was the higher Th1/Th2 ratio (15.39 compared to 6.46 of controls), indicating an attenuated humoral (Th2-mediated) response and consequently the detected deficiency in Ab production.

B cells still remained low in the three groups of CP donors (12.35%, 14.07%, and 11.66% for Ab-negative, non-hospitalized, and hospitalized recovered cases, respectively, compared to 18.74% of controls) and as aforementioned, the reduction of the B cell compartment reached statistical significance (*p* < 0.01; Table 3).

NK cells as a whole, as well as the immature, mature, and memory-like compartments and the mature/memory-like ratio marginally differed from controls, showing that innate immune effectors fully recovered post COVID-19 (Figure 2C). On the contrary, CP donors with severe COVID-19 (hospitalized) comprised the group with the highest levels of NKT cells expressing the marker CD56 (CD3^+^CD56^+^CD16^−^) (8.29% vs. 1.8% of controls), compromising for the slightly lower percentages of the CD3^+^CD56^−^CD16^+^ NKT cell compartment (Figure 2D). Of note, CD3^+^CD56^+^ NKT cells are major contributors in limiting viral spread via the production of high levels of IFN-γ and the expression of CD107, as has been shown in patients infected with HBC and HIV; therefore, their abundant presence in CP donors who required hospitalization may represent a compensatory immune reaction to control their high SARS-CoV-2 load.

Among innate immune cells, the total monocytes in CP donors were similar to the levels of controls; percentages of classical and intermediate monocytes were fully restored, whereas non-classical monocytes still remained high (1.18%, 1.28%, and 0.96% for the three groups), which was similar to the levels recorded in active COVID-19 patients (0.88%). Nevertheless, no statistically significant differences were observed (Figure 2E). Impressively, the mean ratio classical/non-classical monocytes was very increased in CP donors who were hospitalized (1113 vs. 594.1 of controls), although great variability was seen among different individuals. Finally, granulocytes in CP donors were reduced to the levels of controls, but the proportion between activated (CD11b^+^) and resting (CD11b^−^) granulocytes varied with the severity of infection (Figure 2F). Severe COVID-19 recovered CP donors maintained higher percentages of CD11b^+^ granulocytes (and accordingly, a high CD11b^+^/CD11b^−^ ratio), which was followed by CP donors with mild COVID-19 and the Ab-negative group. Similarly, the granulocyte/lymphocyte and granulocyte/CD3^+^ T cell ratios were restored to normal levels.

**Figure 3:**
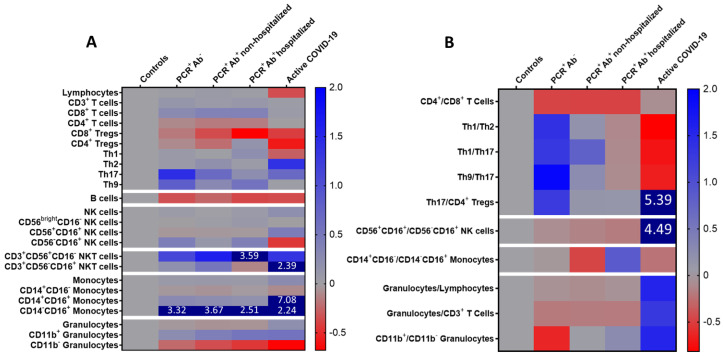
Heatmap of the mean percentages for (**A**) white blood cell populations and (**B**) subpopulation ratios in recovered convalescent plasma (CP) donors and active COVID-19 patients expressed as fold-increase compared to the control group defined as 0. Colors were mapped linearly as in the colored key shown (low in red; high in blue). Numerical values indicate differences exceeding the color scale bar limits.

The aforementioned observations are summarized in the heat maps of Figure 3, which comparatively show all the leukocyte populations studied and ratios calculated. The emerging immune signatures that characterize each group of CP donors are as follows. Ab-negative recovered individuals have high Th17- and Th9-type cells; high Th1/Th2, Th9/Th17 and Th17/CD4^+^ Tregs; low CD11b^+^/CD11b^−^. Recovered individuals with mild COVID-19 have low CD4^+^ Tregs; high non-classical monocytes; low classical/non-classical monocytes. Recovered individuals with severe COVID-19 have low CD8^+^ Tregs; high CD56^+^ NKT cells; low CD11b^−^ granulocytes; low Th1/Th2, Th1/Th17, and Th9/Th17.

## 4. Discussion

Numerous reports so far have analyzed immune responses to SARS-CoV-2 infection during the course of COVID-19 and after a relatively short follow up [25,26,27]; however, data on the long-term effects of the infection on immune responses are sparse [11]. To identify immune variables associated with SARS-CoV-2 infection that may persist following COVID-19 resolution, we evaluated in detail the immune profile of a cohort of CP donors assessed at median 60 days after either the onset of symptoms or a positive PCR result.

The cohort was divided into three groups, comprising CP donors who did not develop a humoral response and accordingly were Ab−, Ab+ CP donors presented with mild disease and thus were not hospitalized, and Ab+ CP donors previously hospitalized due to severe COVID-19 symptoms. Using an in-house-designed 12-color flow cytometry panel, we immunophenotyped 24 distinct cell populations of both the innate (granulocytes, monocytes, NK cells) and the adaptive (T, B, NKT cells) arms of immunity and calculated a series of immune cell ratios to reveal alterations in immune cell composition after COVID-19 resolution. We first analyzed the immune profile of active COVID-19 patients to ensure the performance of our panel; in agreement with previous reports, we detected similar immune cell deficiencies, the most prominent being low T, B, and NK cells, and low granulocytes, as well as increased percentages of Th2-type cells and monocytes [5,6,7,8,10,21,22,23,24].

A common immune signature characterizing COVID-19-recovered donors emerged, irrespective of the humoral response mounted. B cells and CD4^+^ T cells remained at low levels even at 2 months after resolution of the disease, whereas CD3^+^CD56^+^CD16^−^ NKT cells were still higher compared to controls. This shared signature in all CP donors is partly in agreement with a most elegant dynamic COVID-19 immune signature recently reported, although the latter refers to patients with active disease and thus, differences recorded compared to controls are more prominent [28].

In more detail, it is well established that patients infected with SARS-CoV-2 have significantly decreased numbers of NK and CD8^+^ T cells, which display a functionally exhausted phenotype (increased expression of NKG2A and PD-1 and TIM-3, respectively). It is speculated that this decrease is due to NK cell sequestration to infected organs and/or NK cell death. Thus, mature NK cells probably exit the circulation and migrate to the lung where they contribute to local inflammation and injury, while NK cells that remain in PB display an exhausted phenotype (CD56-) that could facilitate viral spread to other organs [6,29,30,31]. Although in our study we did not find a decreased number of NK cells, we observed an increased ratio of mature/memory-like NK cells in patients with COVID-19, implying the reduction of memory NK cells that reflects NK cell exhaustion in their effort to eliminate the virus. When patients recovered, the ratio of mature/memory-like NK cells normalized, irrespective of the severity of the symptoms. It was of interest that CD3^+^CD56^+^CD16^−^ NKT cells were higher in COVID-19 patients than in controls, which is a finding that persisted in CP donors and was more pronounced in those recovered patients with a history of severe disease. The persistence of this state of inflammatory response could imply the persistent presence of the virus or virus segments (viral RNA or protein fragments) that are still antigenic in reservoirs of the body such as the small intestine.

It has been shown that patients infected with SARS-CoV-2 have a high neutrophil count during the severe phase, and the neutrophil to lymphocyte ratio can be used to predict the degree of disease severity in patients with early-stage COVID-19 [32,33]. SARS-CoV-2 infection is also characterized by changes in the frequency of granulocyte subsets and alteration of their functional phenotypes that correlate with COVID-19 severity [34]. Granulocytes and mainly their activated CD11b^+^ subset were significantly increased during the active phase in the group of COVID-19 patients, while the CD11b^−^ subset was diminished. CD11b, which is a subunit of the αMβ2 (CD11bCD18) integrin, has been found to play a critical role in the resolution of the inflammation process [35]. Granulocytes returned to physiological values in all CP donors after a period of approximately 2 months; however, COVID-19 CP donors with severe symptoms who were hospitalized maintained higher percentages of CD11b^+^ granulocytes (and accordingly a high CD11b^+^/CD11b^−^ ratio), followed by CP donors with mild COVID-19 and the Ab-negative group.

Monocytes were decreased in COVID-19 patients compared to recovered patients, which is in line with previous reports [4,36]. This finding may reflect the transition of PB monocytes into tissue macrophages in sites of virus abundance during the active phase of the disease. The SARS-CoV-2-infected monocytes and macrophages that migrate in the tissues can produce large amounts of pro-inflammatory cytokines and chemokines and thus contribute to both local and systemic inflammatory responses. It has also been reported that the CD14^+^CD16^+^ inflammatory intermediate monocyte percentage is higher in COVID-19 patients especially in those with more serious pulmonary complications compared to healthy individuals [36,37]. We confirmed this observation in our active COVID-19 group of patients, but we observed that the proportion of intermediate monocytes returned to normal in all recovered CP donors.

We also confirmed the finding from previous studies that in COVID-19 patients, the total number of lymphocytes is decreased. Lymphopenia has been proposed to serve as a reference index in the diagnosis of SARS-CoV-2 infection [38,39]. It is present in >80% of patients at the time of hospital admission and affects both effector memory and regulatory CD4^+^ T cells [40]. Interestingly, B lymphocyte numbers did not recover after two months in none of the groups of our CP donors, and this was more pronounced in CP donors who did not develop Abs. At the early stages of the disease, the decrease may be attributed to the migration of lymphocytes in the lung, but the persistence of low numbers of B lymphocytes may reflect the exhaustion of humoral responses.

Th1 responses via cytotoxic CD8^+^ T lymphocytes are typically potent in patients with coronavirus infections. In our group of patients, neither CD8^+^ T cells nor Th1 cells differed from controls, which is a finding that could be explained by their depletion at the active stage of the infection mediated by the activation of innate immunity cells, such as NK cells. However, in all CP donors’ groups, CD8^+^ T cells increased and CD4^+^ T cells decreased, leading to a ca. 2-fold reduced CD4^+^/CD8^+^ T cell ratio. A post-treatment decrease in CD8^+^ T cells and increase in CD4^+^/CD8^+^ ratio have been indicated as independent predictors of poor treatment outcomes in COVID-19. Therefore, the recovery of CD8^+^ T cells and the decrease in CD4^+^/CD8^+^ ratio seem to be a characteristic of CP donors. Increased CD8^+^ T cells in conjunction with the persistently high NKT cells and the lower percentages of CD8^+^ Tregs, especially in recovered donors who had severe symptoms and were hospitalized, could further strengthen the hypothesis that the prolonged (60 days after the infection) presence of CD8^+^ effectors probably reflects the necessity to modulate anti-viral responses against parts of the virus hidden in sanctuaries in this group of donors [30,41].

Th2 responses were increased in patients depicting the increasing humoral response in order to produce Abs that could neutralize the virus. The ratio Th1/Th2 was decreased in patients but was restored in the two groups of recovered CP donors who produced Abs. The higher Th1/Th2 ratio that was noticed in the PCR+Abs− donors’ group may reflect an attenuated humoral (Th2-mediated) response that consequently led to the detected deficiency in Ab production.

Negative immune regulation and a skewing toward Th17 responses have been mainly described in COVID-19 patients who develop a hyperinflammatory response. The release of cytokines such as IL-17 and GM-CSF can exacerbate responses through the downregulation of Tregs, promotion of neutrophil migration, and simultaneous induction of Th2 responses. Tregs can inhibit multiple immune cells, such as CD8^+^, CD4^+^ T cells, monocytes, NK cells, as well as B cells controlling inappropriate immune responses. Tregs can interfere with CD8^+^ T cell and CD4^+^ T cell proliferation and survival through competing for IL-2, inhibit antigen-presenting cell maturation, and eliminate effector cells through secreting cytokines such as TGF-β and IL-10. It has been hypothesized that the modulation of Tregs may help prevent COVID-19 transition from mild to severe and even to treat severe COVID-19 patients [36,42].

We found reduced CD4^+^ Tregs in COVID-19 patients and an increased ratio of Th17/Tregs. This finding could be an indication of Tregs’ consumption in order to block unwanted autoimmune and immune responses in the active phase of the disease that may lead to insufficient regulation of pro-inflammatory immune responses and can further aggravate hyper-inflammation. In all groups of CP donors, Th1-, Th2-type, and CD4^+^ Tregs were restored, while a Th17- and Th9-polarized immunity was recorded in recovered individuals that did not generate Abs. Th9 responses mediated by IL-9 secretion have been reported to participate in tissue inflammation and immune-mediated diseases ranging from autoimmunity to asthma [43]. This skewed immune response (highly increased Th9/Th17 ratio) could be associated with the persistence of a generalized inflammatory reaction (mediated by Th17 cells) being more intense in the lung (mediated by Th9 cells) of Ab-negative recovered individuals.

The limitations of our study pertain to the relatively small number of hospitalized COVID-19 patients that was evaluated (*n* = 11) and that all analyses were performed in PB samples. Alveolar samples could better demonstrate the immune responses generated, especially during the acute phase of the disease. However, our study provides important information on phenotypic immune signatures persisting after SARS-CoV-2 infection, highlighting differences between Ab-negative, mild, and severe COVID-19 cases. The confirmation of our findings in larger number of patients and follow-up samples could enable the identification of immunophenotypic patterns that could predict the outcome of the disease and the development of humoral responses. The latter is particularly important for the sustained immune responses needed for the successful development of vaccines against SARS-CoV-2.

## 5. Conclusions

In conclusion, the immune profile of SARS-CoV-2 recovered CP donors, at least within 2 months after either the onset of symptoms or a positive PCR result, shows characteristic alterations in innate (monocytes and granulocytes) but most importantly in adaptive (T, B, and NKT) immune cells. These persistent changes seem to be related to the degree of activation of the humoral arm of immunity against the virus as well as to the severity of the disease.

## Figures and Tables

**Table 1:** Demographic and clinical characteristics of COVID-19 related groups.

	Controls (*n* = 10)	PCR+ Ab− Donors (*n* = 12)	PCR+ Ab+ Non-Hospitalized CP Donors (*n* = 38)	PCR+ Ab+ Hospitalized CP Donors (*n* = 24)	Active COVID-19 Patients (*n* = 11)
**Male gender (percentage)**	6 (60%)	5 (41.7%)	19 (50%)	16 (66.7%)	8 (72.7%)
**Age in years, mean (range)**	44.4 (16–82)	41.7 (30–60)	43.1 (18–72)	58.6 (37–78)	60.5 (40–82)
**Symptoms ≥ 2 (percentage)**	0	8 (66.7%)	31 (81.6%)	22 (91.7%)	5 (50%)
**IgA, mean**	0.55	0.64	5.55	3.34	N/A
**IgG, mean**	0.29	0.46	7.32	4.90	N/A

N/A: not applicable.

**Table 2:** Immune subsets analyzed and their respective phenotypes.

Immunesubset	Phenotype
White blood cells (WBCs)	CD45^+^
CD3^+^ T cells	CD45^+^ CD3^+^ SSC^low^
CD8^+^ cytotoxic T cells	CD45^+^ CD3^+^ CD8^+^ SSC^low^
CD8^+^ regulatory T cells (CD8^+^ Tregs)	CD45^+^ CD3^+^ CD8^+^ CD25^high^ SSC^low^
CD4^+^ helper T cells	CD45^+^ CD3^+^ CD4^+^ SSC^low^
CD4^+^ regulatory T cells (CD4^+^ Tregs)	CD45^+^ CD3^+^ CD4^+^ CD25^high^ SSC^low^
Th1-type	CD45^+^ CD3^+^ CD4^+^ CD183^+^ SSC^low^
Th2-type	CD45^+^ CD3^+^ CD4^+^ CD194^+^ SSC^low^
Th17-type	CD45^+^ CD3^+^ CD4^+^ CD194^+^ CD196^+^ SSC^low^
Th9-type	CD45^+^ CD3^+^ CD4^+^ CD196^+^ SSC^low^
B cells	CD45^+^ CD3- CD16^−^ CD56^−^ SSC^low^
Mature NK cells	CD45^+^ CD3- CD56^+^ CD16^+^ SSC^low^
Immature NK cells	CD45^+^ CD3^−^ CD56^bright^ CD16^−^ SSC^low^
Memory-like NK cells	CD45^+^ CD3^−^ CD56^−^ CD16^+^ SSC^low^
CD56^+^ CD16^−^ NKT cells	CD45^+^ CD3^+^ CD56^+^ CD16^−^ SSC^low^
CD56^−^ CD16^+^ NKT cells	CD45^+^ CD3^+^ CD56^−^ CD16^+^ SSC^low^
Classical monocytes	CD45^+^ CD14^+^ CD16^−^ SSC^int^
Intermediate monocytes	CD45^+^ CD14^+^ CD16^+^ SSC^int^
Non-classical monocytes	CD45^+^ CD14^−^ CD16^+^ SSC^int^
CD11b^+^ activated granulocytes	CD45^+^ CD11b^+^ SSC^high^
CD11b^−^ granulocytes	CD45^+^ CD11b^−^ SSC^high^

NK: Natural killer; SSC: Side scatter; bright: bright expression; int: intermediate expression; low: low expression.

**Table 3:** Mean percentages of immune cell populations and cell ratios with the relative statistical significance of differences between groups.

Immune Subset/ Ratio (% of Parent Population)	Controls ^a^	PCR+Ab− Donors ^a^	PCR+Ab+ Non-Hospitalized CP Donors ^a^	PCR+Ab+ Hospitalized CP Donors ^a^	Active COVID-19 Patients ^a^	*p* Value ^b^
Lymphocytes (% of WBCs)	31.41 ± 7.78 **	32.35 ± 8.44 **	33.40 ± 9.12 **	32.21 ± 9.42 **	20.31 ± 10.10 *	0.002
CD3^+^ T cells (% of lymphocytes)	67.07 ± 8.61	74.73 ± 4.85	71.88 ± 6.97	74.39 ± 7.24	69.69 ± 12.05	0.198
CD8^+^ T cells (% of CD3^+^ T cells)	24.38 ± 8.51	31.02 ± 7.27	33.49 ± 10.11	33.49± 10.60	25.66 ± 11.87	0.030
CD8^+^ Tregs (% of CD8^+^ T cells)	7.29 ± 8.24	6.07 ± 4.97	4.67 ± 6.21	2.35 ± 3.13	4.23 ± 3.16	0.199
CD4^+^ T cells (% of CD3^+^ T cells)	71.36 ± 11.28 **	64.11 ± 7.64	60.11 ± 10.15 *	60.75 ± 11.39	70.51 ± 13.21 **	0.006
CD4^+^ Tregs (% of CD4^+^ T cells)	2.05 ± 0.90 **	1.84 ± 0.90	1.59 ± 0.69	2.37 ± 1.30**	0.86 ± 0.63 *	<0.001
Th1 (% of CD4^+^ T cells)	26.77 ± 7.96	29.06 ± 8.18 **	27.31 ± 7.55 **	32.51 ± 8.55 **	19.40 ± 5.74*	<0.001
Th2 (% of CD4^+^ T cells)	7.67 ± 3.60 **	8.27 ± 8.91 **	9.16 ± 7.61 **	8.07 ± 4.71 **	18.32 ± 6.10 *	0.001
Th17 (% of CD4^+^ T cells)	1.73 ± 0.96	4.22 ± 6.58	2.88 ± 4.58	2.14 ± 1.49	2.92 ± 2.72	0.925
Th9 (% of CD4^+^ T cells)	11.57 ± 4.10	21.08 ± 12.63	15.00 ± 10.53	18.10 ± 12.66	11.89 ± 8.47	0.130
B cells (% of lymphocytes)	18.74 ± 5.60 *	12.35 ± 2.06 **	14.07 ± 4.56 **	11.66 ± 5.60 **	11.97 ± 4.55 **	0.002
NK cells (% of lymphocytes)	12.14 ± 6.13	12.85 ± 7.35	12.15 ± 6.59	12.98 ± 5.41	14.84 ± 11.34	0.930
Immature NK cells (% of NK cells)	0.69 ± 0.32	0.68 ± 0.28	0.71 ± 0.62	0.75 ± 0.55	0.68 ± 0.41	0.913
Mature NK cells (% of NK cells)	8.84 ± 5.05	8.47 ± 5.25	8.54 ± 5.33	8.57 ± 5.00	12.71 ± 10.47	0.803
Memory-like NK cells (% of NK cells)	2.61 ± 1.20	3.70 ± 3.40	2.90 ± 1.38 **	3.65 ± 1.66 **	1.44 ± 1.66 *	0.003
CD3^+^CD56^+^CD16^−^ NKT cells (% of CD3^+^ cells)	1.80 ± 2.05 *	3.82 ± 2.28	4.70 ± 3.71 **	8.29 ± 6.53 **	4.22 ± 4.68	<0.001
CD3^+^CD56^-^CD16^+^ NKT cells (% of CD3^+^ cells)	0.19 ± 0.13	0.23 ± 0.31	0.29 ± 0.38	0.17 ± 0.18	0.64± 1.14	0.767
Monocytes (% of WBCs)	3.52 ± 0.66	3.82 ± 1.12	3.87 ± 1.16	3.81 ± 1.19	4.41 ± 1.36	0.481
Classical monocytes (% of monocytes)	95.04 ± 3.14	91.63 ± 11.23	89.80 ± 13.21	92.73 ± 6.00	86.25 ± 9.16	0.046
Intermediate monocytes (% of monocytes)	0.81 ± 0.64 **	1.04 ± 0.88 **	1.00 ± 0.89 **	0.96 ± 0.91 **	6.57 ± 6.68 *	<0.001
Non-classical monocytes (% of monocytes)	0.27 ± 0.24	1.18 ± 2.40	1.28 ± 2.08	0.96 ± 1.38	0.88 ± 1.30	0.189
Granulocytes (% of WBCs)	56.84 ± 8.56 **	54.77 ± 9.55 **	53.26 ± 9.48 **	53.48 ± 9.89 **	69.00 ± 12.30 *	<0.001
CD11b^+^ Granulocytes (% of granulocytes)	46.12 ± 34.14	57.34 ± 23.56	65.53 ± 26.21	70.89 ± 19.45	72.33 ± 18.24	0.156
CD11b^−^ Granulocytes (% of granulocytes)	47.29 ± 27.52	36.13 ± 20.28	30.50 ± 24.68	26.18 ± 18.71	15.74 ± 9.40	0.037
CD4^+^/CD8^+^ T cells	4.51 ± 3.66	2.20 ± 0.66	2.17 ± 1.67	2.16 ± 12.14	3.77 ± 2.71	0.025
Th1/Th2	6.46 ± 9.38 **	15.39 ± 20.67 **	7.55 ± 11.08 **	5.67 ± 4.10 **	1.19 ± 0.53 *	<0.001
Th1/Th17	41.54 ± 72.48	95.54 ± 138.30	74.65 ± 168.90	36.77 ± 49.67	11.63 ± 7.87	0.324
Th9/Th17	14.33 ± 20.89	41.82 ± 61.57 **	17.87 ± 30.10**	12.83 ± 11.00 **	4.72 ± 1.38 *	0.020
Th17/CD4^+^ Tregs	1.42 ± 1.54	3.23 ± 4.11	1.61 ± 1.84	1.59 ± 2.37	9.09 ± 19.41	0.070
Mature/memory-like NK cells	3.55 ± 1.35	3.21 ± 2.86 **	3.03 ± 1.80 **	2.89 ± 2.22 **	19.47 ± 30.79 *	<0.001
Classical/non-classical monocytes	594.1 ± 395.4	567.5 ± 909.5	315.7 ± 354.1	1113.0± 2724.1	458.6 ± 552.7	0.199
Granulocytes/lymphocytes	2.02 ± 1.15	1.87 ± 0.79	1.82 ± 0.88 **	1.88 ± 0.84 **	5.16 ± 5.09 *	0.007
Granulocytes/CD3^+^ T cells	3.14 ± 2.20	2.53 ± 1.13 **	2.56 ± 1.30 **	2.54 ± 1.17 **	7.19 ± 5.88 *	0.006
CD11b^+^/CD11b^−^ granulocytes	6.40 ± 13.18	2.36 ± 1.73	6.63 ± 8.08	8.03 ± 14.83	16.26 ± 35.84	0.053

^a^ mean percentage ± standard deviation from *n* = 10 for controls; *n* = 12 for PCR+Ab− CP donors; *n* = 38 for PCR+Ab+ non-hospitalized CP donors; *n* = 24 for PCR+Ab+ hospitalized CP donors; *n* = 11 for active COVID-19 patients. ^b^ *p* values in bold denote statistically significant differences (one-way ANOVA); * vs. **, significant differences per immune subset with Holm–Sidak post-hoc test.

## Supplementary Materials

The following are available online at https://www.mdpi.com/1999-4915/13/1/26/s1, Figure S1: (A) Lymphocytes, monocytes and granulocytes were gated on their characteristic side scatter patterns and CD45 expression. Lymphocytes were classified based on CD3 expression to identify T cells, then divided into CD4^+^ and CD8^+^ T cells, prior to separation into their subsets of T regulatory expressing CD8 or CD4. The CD4^+^ subset was further categorized into Th1, Th2, Th9 and Th17. CD3^–^ lymphocytes were characterized as NK cells or B cells based on expression of CD16 and CD56. NK subpopulations were gated based on the expression pattern of CD56 and CD16. CD3^+^ lymphocytes were characterized as NKT cells based on expression of CD16 and CD56. Monocytes were subdivided by expression of CD14 and CD16. Granulocytes were analyzed for expression of the CD11b marker. (B) Population hierarchy tree for the analysis of the populations and the subpopulations of the samples. (C) Expression of CD196 on Th1 and Th2 cells (top 2 rows), of CD194 on Th9 cells (middle) and of CD183 on Th17 cells (bottom). Monocytes which are negative for CD196, CD194 and CD183 are shown with blue color., Figure S2: Distribution of (A) CD8^+^ T and (B) CD4^+^ T cells as percentage of CD3^+^ T cells; (C) CD4^+^ Tregs, (D) Th1 and (E) Th2 cells as percentage of CD4^+^ T cells; (F) memory-like NK cells as percentage of lymphocytes; (G) CD3^+^CD56^+^CD16^−^ subpopulation of NKT cells as percentage of CD3^+^ cells; (H) classical (CD14^+^CD16^−^) and (I) intermediate monocytes (CD14^+^CD16^+^) as percentage of total monocytes; and (J) CD11b^−^ granulocytes as percentage of total granulocytes, in healthy donors, the three groups of recovered CP donors and active COVID-19 patients. *p* values shown were calculated with one-way ANOVA.
