# Supplementary material for: Peripheral Blood Immune Profiling of Convalescent Plasma Donors Reveals Alterations in Specific Immune Subpopulations Even at 2 Months Post SARS-CoV-2 Infection
Source: Viruses. 2020 Dec 25;13(1):26. doi: 10.3390/v13010026 (PMC7824046; doi:10.3390/v13010026)
Supplement: Supplementary file 1 [file viruses-13-00026-s001.pdf]

viruses

MDPI

Supplemental figures

A

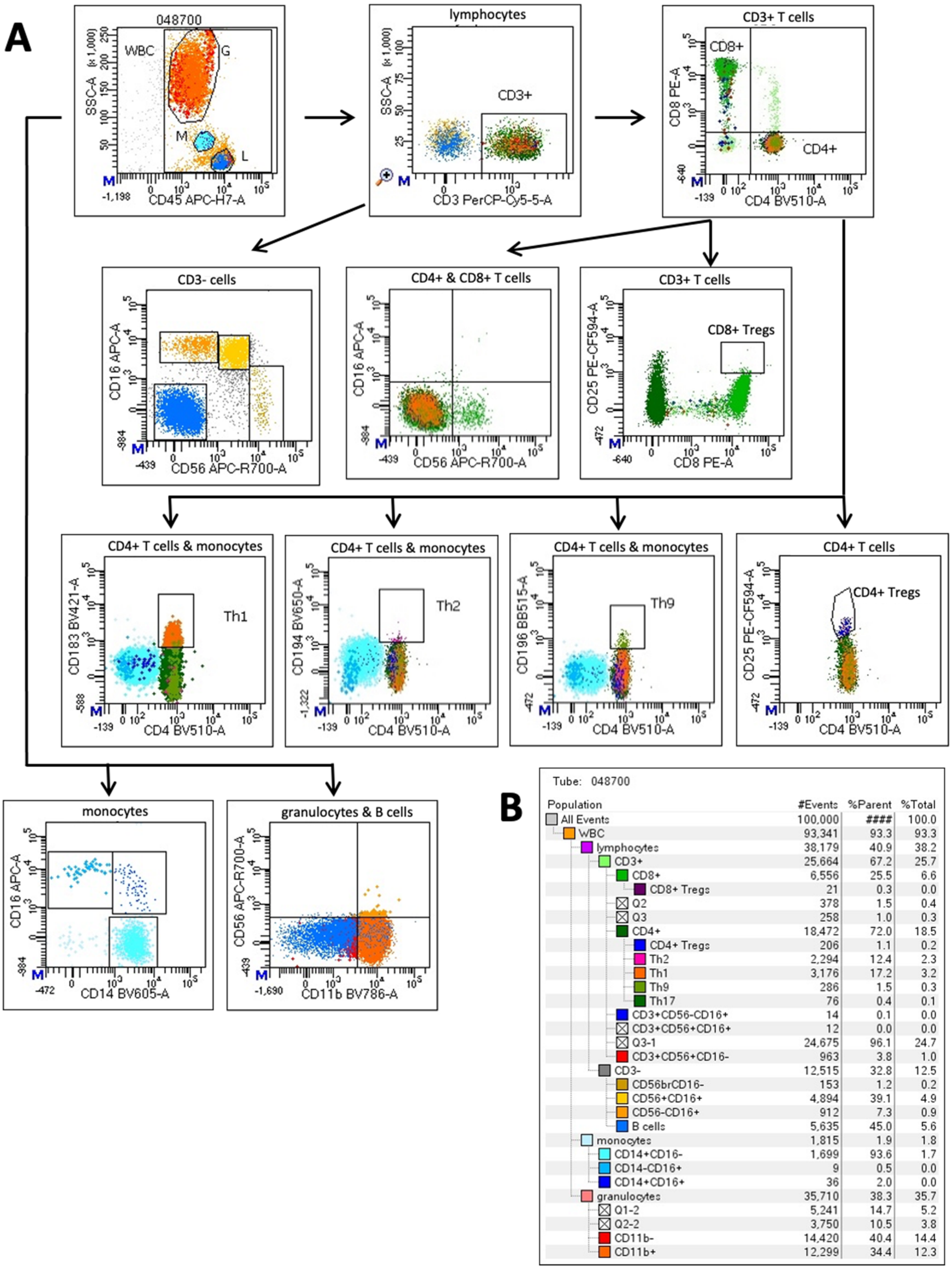

B

Tube: 048700

Population	#Events	%Parent	%Total
All Events	100,000	####	100.0
WBC	93,341	93.3	93.3
lymphocytes	38,179	40.9	38.2
CD3+	25,664	67.2	25.7
CD8+	6,556	25.5	6.6
CD8+ Tregs	21	0.3	0.0
Q2	378	1.5	0.4
Q3	258	1.0	0.3
CD4+	18,472	72.0	18.5
CD4+ Tregs	206	1.1	0.2
Th2	2,294	12.4	2.3
Th1	3,176	17.2	3.2
Th9	286	1.5	0.3
Th17	76	0.4	0.1
CD3+CD56-CD16+	14	0.1	0.0
CD3+CD56+CD16+	12	0.0	0.0
Q3-1	24,675	96.1	24.7
CD3+CD56+CD16-	963	3.8	1.0
CD3-	12,515	32.8	12.5
CD56brCD16-	153	1.2	0.2
CD56+CD16+	4,894	39.1	4.9
CD56-CD16+	912	7.3	0.9
B cells	5,635	45.0	5.6
monocytes	1,815	1.9	1.8
CD14+CD16-	1,699	93.6	1.7
CD14-CD16+	9	0.5	0.0
CD14+CD16+	36	2.0	0.0
granulocytes	35,710	38.3	35.7
Q1-2	5,241	14.7	5.2
Q2-2	3,750	10.5	3.8
CD11b-	14,420	40.4	14.4
CD11b+	12,299	34.4	12.3

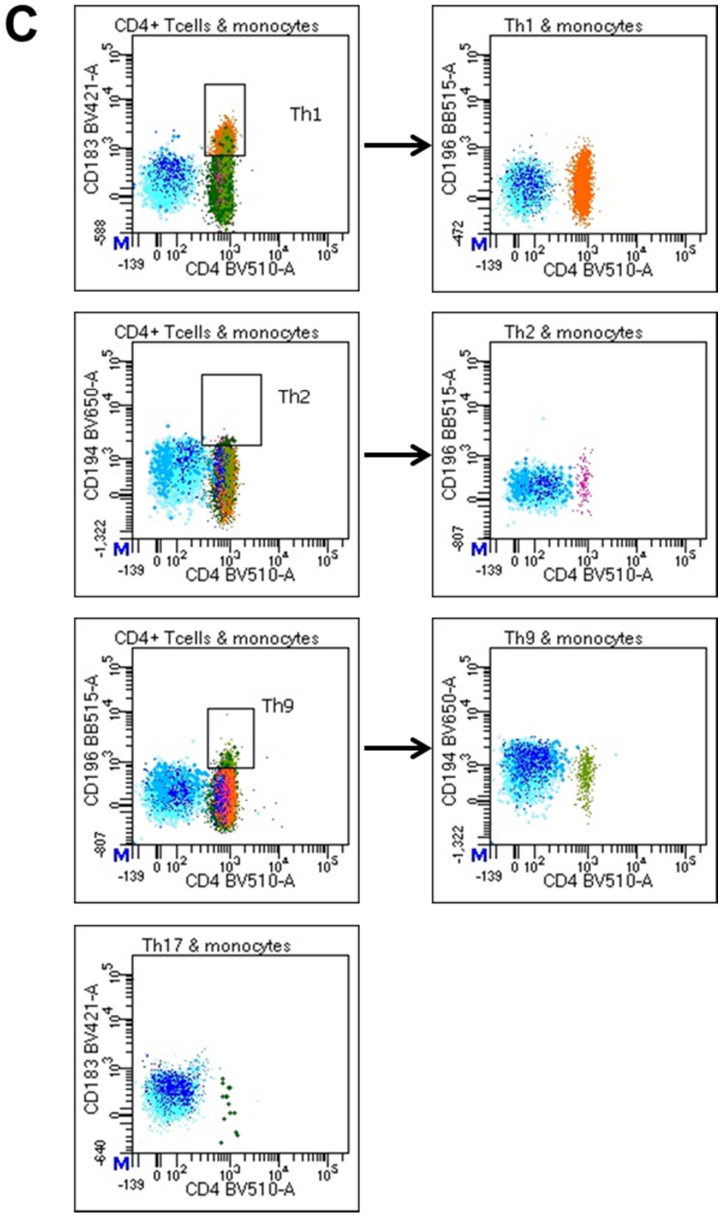

Figure 1. (**A**) Lymphocytes, monocytes and granulocytes were gated on their characteristic side scatter patterns and CD45 expression. Lymphocytes were classified based on CD3 expression to identify T cells, then divided into CD4+ and CD8+ T cells, prior to separation into their subsets of T regulatory expressing CD8 or CD4. The CD4+ subset was further categorized into Th1, Th2, Th9 and Th17. CD3– lymphocytes were characterized as NK cells or B cells based on expression of CD16 and CD56. NK subpopulations were gated based on the expression pattern of CD56 and CD16. CD3+ lymphocytes were characterized as NKT cells based on expression of CD16 and CD56. Monocytes were subdivided by expression of CD14 and CD16. Granulocytes were analyzed for expression of the CD11b marker. (**B**) Population hierarchy tree for the analysis of the populations and the subpopulations of the samples. (**C**) Expression of CD196 on Th1 and Th2 cells (top 2 rows), of CD194 on Th9 cells (middle) and of CD183 on Th17 cells (bottom). Monocytes which are negative for CD196, CD194 and CD183 are shown with blue color.

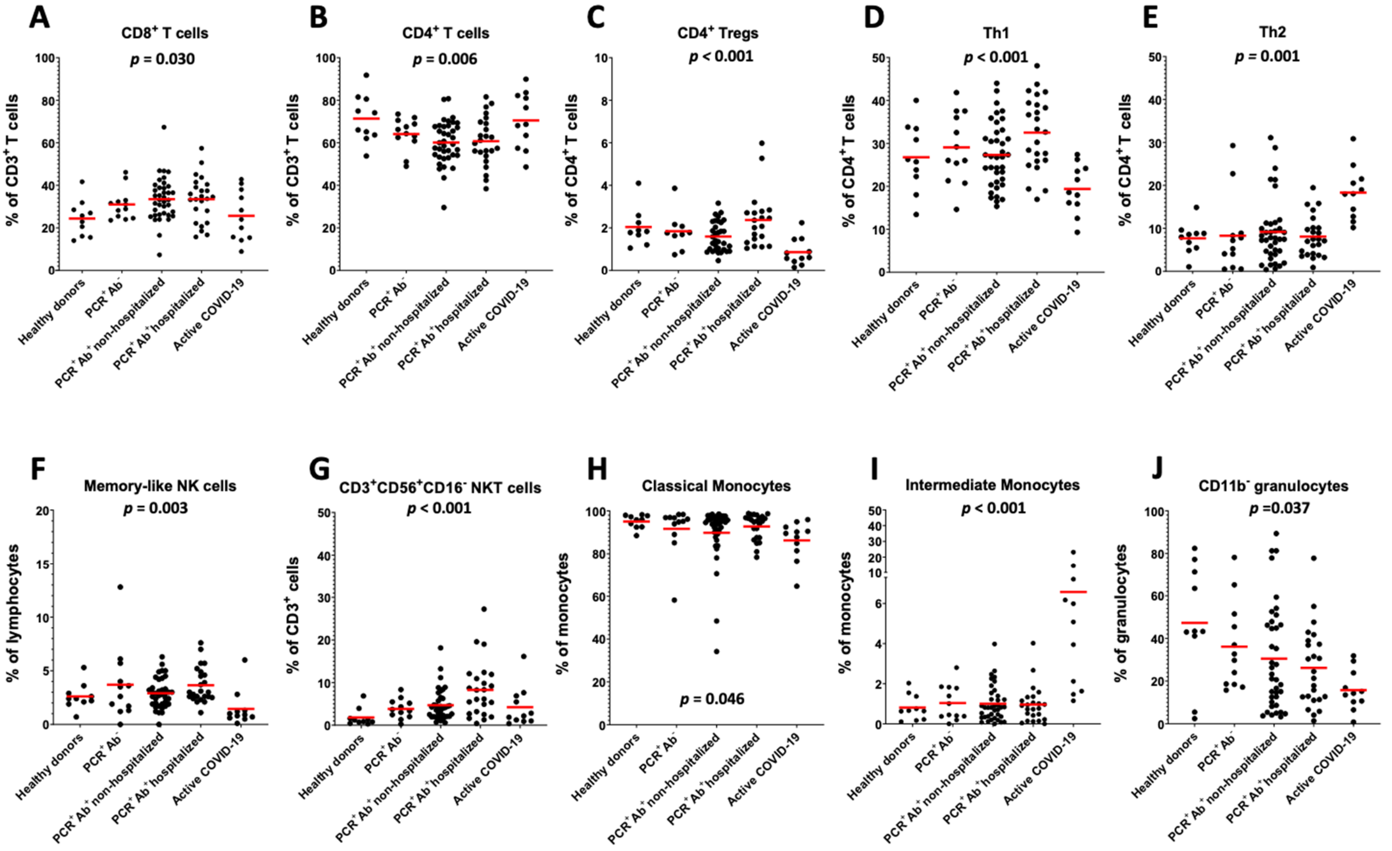

Figure 2. Distribution of (**A**) CD8+ T and (**B**) CD4+ T cells as percentage of CD3+ T cells; (**C**) CD4+ Tregs, (**D**) Th1 and (**E**) Th2 cells as percentage of CD4+ T cells; (**F**) memory-like NK cells as percentage of lymphocytes; (**G**) CD3+CD56+CD16- subpopulation of NKT cells as percentage of CD3+ cells; (**H**) classical (CD14+CD16-) and (**I**) intermediate monocytes (CD14+CD16+) as percentage of total monocytes; and (**J**) CD11b- granulocytes as percentage of total granulocytes, in healthy donors, the three groups of recovered CP donors and active COVID-19 patients. P values shown were calculated with one-way ANOVA.
